# Association of elevated circulating iron with bone mineral density and bone turnover markers in postmenopausal Saudi women with and without osteoporosis

**DOI:** 10.1515/med-2026-1507

**Published:** 2026-08-31

**Authors:** Afnan Ghaleb, Saba Abdi, Sobhy Yakout, Syed D. Hussain, Shaun Sabico, Omar S. Al-Attas, Nasser M. Al-Daghri

**Affiliations:** Chair for Biomarkers of Chronic Diseases, Department of Biochemistry, College of Science, King Saud University, Riyadh, Saudi Arabia

**Keywords:** elevated circulating iron, bone mineral density, bone turnover markers, transferrin saturation, postmenopausal women, Saudi Arabia

## Abstract

**Objectives:**

To explore the associations of serum iron indices with bone mineral density (BMD) and bone turnover markers (BTMs) in postmenopausal Saudi women with and without osteoporosis.

**Methods:**

This cross-sectional study included 184 postmenopausal women aged ≥45 years (75 osteoporotic and 109 controls). Anthropometric and biochemical parameters were assessed. Serum iron, transferrin, total iron-binding capacity (TIBC), and transferrin saturation (TSAT) were measured. Bone-specific alkaline phosphatase (BsALP), osteocalcin, and osteopontin were evaluated. Participants were classified based on serum iron levels (50–170 μg/dL [normal circulating iron] and >170 μg/dL [elevated circulating iron]). BMD was measured at the lumbar spine and femur by dual-energy X-ray absorptiometry (DXA).

**Results:**

Osteoporotic women had significantly lower BMD at the spine and femur (p<0.001). Elevated circulating iron was observed in 54.3 % of participants. Serum iron weakly correlated with osteocalcin (r=0.17, p=0.02) and osteopontin (r=0.17, p=0.049) only in unadjusted analyses. All observed associations were not supported by the primary adjusted analysis.

**Conclusions:**

The primary adjusted analysis showed no statistically significant association between elevated circulating iron and osteoporosis risk. The observed weak associations between serum iron and selected bone turnover markers should be considered exploratory. Findings do not support an independent association between elevated circulating iron and postmenopausal osteoporosis.

## Introduction

Osteoporosis is the most common bone disorder, characterized by reduced bone mass and disruption of the micro-architecture [1], 2]. Osteoporosis results in low bone strength and an increased risk of fragility fractures, which most commonly occur in the spine, wrist, hip, pelvis, and humerus. The risk of these fractures increases gradually with age and is particularly common among elderly people, especially those over 75 years [2], [3], [4]. Globally, more than 200 million people suffer from osteoporosis [5]. The prevalence of osteoporosis in the elderly has been reported to be 21.7 % (women: 35.3 %; men: 12.5 %), with the highest prevalence of 24.3 % in Asia [6]. In the Eastern Mediterranean region, the prevalence was 24.4 % [6]. Among Eastern Mediterranean countries, Saudi Arabia exhibited the highest frequency of osteoporosis at 32.7 %, while Kuwait recorded the lowest at 15.1 % [6]. Approximately 50 % of postmenopausal women aged over 50 years are affected by osteoporosis [7], 8]. During aging, peri- and postmenopausal women are vulnerable to type 1 osteoporosis due to declining estrogen levels [9].

Iron is an essential trace element with an important role in biological systems [10]. It participates in haemoglobin synthesis, mitochondrial function, and enzymatic activity [11]. Despite the well-established global burden of postmenopausal osteoporosis, data from Middle Eastern and Gulf Cooperation Council (GCC) populations remain substantially under-represented in the literature. Saudi women are exposed to several factors that may influence bone health, including the high prevalence of Vitamin D deficiency and population-specific environmental and lifestyle characteristics [12], 13]. Furthermore, the relationship between circulating iron and bone metabolism in this specific ethnic group has not been systematically evaluated [14]. Addressing this evidence gap is critical for developing region-specific clinical guidelines for the prevention and management of postmenopausal osteoporosis in Saudi Arabia and the broader Middle East [15].

Disturbance of bone homeostasis results from an imbalance between osteoblast-mediated bone formation and osteoclast-mediated bone resorption, leading to osteopenia, bone loss, and ultimately osteoporosis [16]. Excess iron accumulation in body tissues, as observed in hemochromatosis, adversely affects many organs including the liver, heart, and endocrine glands [15], 17]. Experimental evidence has suggested an association between excess iron and impaired bone remodelling; however, whether elevated circulating iron is independently associated with osteoporosis in humans remains uncertain and requires confirmation in prospective studies [18].

The association between iron content and menopause was first recognized in 2009 by Jian et al., who reported that increases in serum ferritin concentration, together with declining estrogen levels, occur during the perimenopausal period in women around 45 years of age [19]. This hypothesis has been explored in subsequent clinical studies [19], 20]. These studies suggested that increased serum ferritin may be associated with accelerated bone loss, reduced bone mineral density, and impaired bone strength [21], [22], [23]. The association between elevated circulating iron and osteoporosis in postmenopausal women has been reported in diverse previous studies [23], [24], [25], [26], [27]. However, the cellular mechanisms explaining the influence of iron on bone are not yet completely clear [28]. There is limited information regarding the association between elevated circulating iron and bone turnover markers (BTMs) in postmenopausal women with osteoporosis [29].

*In vitro* data suggest that iron may be associated with bone damage through its role in the Fenton reaction, catalysing the production of reactive oxygen species (ROS) that may inhibit osteoblast differentiation and stimulate osteoclast activity [30], 31]. These mechanistic hypotheses require confirmation in longitudinal human studies before causal conclusions can be drawn [32].

Saudi Arabia has a higher prevalence of osteoporosis in postmenopausal women compared to other Middle Eastern countries [6], 33]. In 2022, a study in Riyadh by Alkhunizan et al. estimated the prevalence of osteoporosis among older females aged ≥60 years to be 83.1 % [34]. Few studies have investigated the association between elevated circulating iron and bone health among postmenopausal Arab women [26]. The purpose of the current study was to investigate the associations of serum iron indices with osteoporosis status, bone mineral density, and bone turnover markers in postmenopausal Saudi women.

## Methods

This study was reported in accordance with the Strengthening the Reporting of Observational Studies in Epidemiology (STROBE) guidelines for cross-sectional studies [35].

### Study design and participants

A total of 184 postmenopausal women aged ≥45 years participated in this cross-sectional study. The participant selection process is shown in Supplementary Figure S1. The control group (without osteoporosis) comprised 109 participants, while the patient group (with osteoporosis) comprised 75 participants. All participants were recruited from several hospitals in Riyadh, Kingdom of Saudi Arabia.

The World Health Organization (WHO) defines osteoporosis as a BMD T-score ≤ −2.5 SD below the mean peak bone mass of a young healthy adult woman [1]. BMD was measured at the lumbar spine (L1–L4) and dual femur (left) using dual-energy X-ray absorptiometry (DXA) (Hologic QDR 2000 Inc., Waltham, MA, USA) for the diagnosis of osteoporosis. Women with chronic diseases including malignancy, acute inflammation, and a history of kidney, heart, or liver disease were excluded. No formal sample size or power calculation was performed prior to this study, which represents a limitation of the current work.

Anthropometric measurements, including mean blood pressure (BP), height, weight, waist-to-hip ratio, and body mass index (BMI), were obtained. An osteoporosis registry form was completed for all subjects. This study was approved by the Research Ethics Committee, College of Science, King Saud University, Riyadh, Saudi Arabia (Approval No. 8/25/454,266) on 30 September 2013. The study was conducted in accordance with the Declaration of Helsinki (2013 revision). Informed consent was obtained from all individuals included in this study.

### Sample analysis and laboratory measurements

Fasting glucose (after 8–12 h), calcium (Ca), albumin (ALB), and lipid profile including total cholesterol (CHL), high-density lipoprotein cholesterol (HDL-CHL), triglycerides (TRIG), and low-density lipoprotein cholesterol (LDL-CHL) were measured using an automated analyzer (Konelab, Thermo Fisher Scientific, Vantaa, Finland). Serum 25-hydroxyvitamin D was measured by enzyme-linked immunosorbent assay (ELISA) (IDS Ltd., Boldon Colliery, Tyne & Wear, UK). N-telopeptide cross-linked of type I collagen (NTx), beta-carboxyterminal telopeptide of type I collagen (β-CrossLaps), N-terminal propeptide of type I procollagen (P1NP), and BsALP were measured by electrochemiluminescence immunoassay using the Cobas e411 automated analyzer (Roche Diagnostics, Mannheim, Germany). Insulin, osteoprotegerin, osteocalcin, osteopontin, and sclerostin were determined using the Luminex Multiplex Assay system (Luminex Inc., Austin, TX, USA).

### Assay performance

All assays were performed according to the manufacturers’ instructions, and quality control samples were included in each run. The reported intra-assay and inter-assay coefficients of variation (CVs) were based on manufacturer specifications provided in the respective assay manuals. BsALP: intra-assay CV <5.0 %, inter-assay CV <7.5 %; Osteocalcin: intra-assay CV <4.8 %, inter-assay CV <8.2 %; Osteopontin: intra-assay CV <5.5 %, inter-assay CV <9.0 %; NTx: intra-assay CV <4.2 %, inter-assay CV <7.8 %; β-CTX: intra-assay CV <4.9 %, inter-assay CV <8.5 %; P1NP: intra-assay CV <3.8 %, inter-assay CV <7.1 %; OPG: intra-assay CV <5.8 %, inter-assay CV <9.3 %; Sclerostin: intra-assay CV <4.5 %, inter-assay CV <8.0 %. Serum iron and TIBC: intra-assay CV <2.0 %, inter-assay CV <4.0 % (spectrophotometric method using a SpectraMax M5 microplate reader, Molecular Devices, San Jose, CA, USA).

### Determination of iron indices

Serum iron concentration and total iron-binding capacity (TIBC) were determined by spectrophotometry using a SpectraMax M5 microplate reader (Molecular Devices, San Jose, CA, USA). Serum iron and TIBC were reported in µg/dL. Transferrin (TRf) concentration was measured using a commercially available ELISA kit (R&D Systems, Minneapolis, MN, USA) and expressed in mg/L according to the manufacturer’s calibration and recommended reporting units. Transferrin saturation (TSAT) was calculated as [36].

$$\text{TSAT }\left(\%\right)=\left[\text{Serum iron }\left(µg/\text{dL}\right)/\text{TIBC }\left(µg/\text{dL}\right)\right]\times 100$$

Participants were classified into two groups based on serum iron levels: normal circulating iron (50–170 μg/dL) and elevated circulating iron (>170 μg/dL) , using the upper limit of the local laboratory reference range as an operational threshold. This threshold does not represent a validated clinical definition of iron overload, which is conventionally based on transferrin saturation (TSAT ≥45 %) and/or elevated serum ferritin. A sensitivity analysis using TSAT ≥45 % yielded directionally similar findings; however, only 12 participants met this criterion, limiting statistical interpretation.

### Statistical analysis

Statistical analyses were performed using SPSS version 16.0 (IBM Corp., Chicago, IL, USA; 2007). Normality of continuous variables was assessed using the Shapiro-Wilk test. Normally distributed variables are presented as mean ± SD and compared between groups using independent samples t-tests; non-normally distributed variables are presented as median (IQR) and compared using Mann-Whitney U tests. Categorical variables are presented as frequencies and percentages and compared using chi-square tests. Pearson correlation coefficients were calculated for normally distributed variables and Spearman correlation coefficients for non-normally distributed variables. Binary logistic regression was used to examine the adjusted association between serum iron status and osteoporosis, with age and BMI as pre-specified covariates; model calibration was assessed using the Hosmer-Lemeshow test. All analyses were exploratory and hypothesis-generating. Given the large number of comparisons (>25 variables), no correction for multiple comparisons was applied, and all results should be interpreted with caution as potentially subject to type I error. A post-hoc power analysis (G*Power 3.1) indicated that with n=75 osteoporotic and n=109 controls, the study had approximately 62 % power to detect a medium effect size (OR≈2.0) at α=0.05 (two-tailed), indicating that the study was underpowered for definitive conclusions. A two-tailed p-value <0.05 was considered nominally significant for exploratory purposes.

## Results

A total of 184 postmenopausal Saudi women aged ≥45 years were enrolled in this cross-sectional study: 109 controls (without osteoporosis) and 75 women with osteoporosis, defined as a DXA T-score ≤ −2.5 at the lumbar spine or left femur. Of the total cohort, 54.3 % (n=100) had elevated serum iron (>170 μg/dL) and 45.7 % (n=84) had normal serum iron (50–170 μg/dL). Figure 1 illustrates this overall distribution of iron status across the study population. Figure 2 further stratifies iron status by osteoporosis group: among controls, 56.9 % (n=62) had elevated iron compared with 50.7 % (n=38) of osteoporotic women; this difference was not statistically significant (χ²=0.67, p=0.413), indicating that the crude prevalence of elevated circulating iron was comparable between groups.

**Figure 1:**
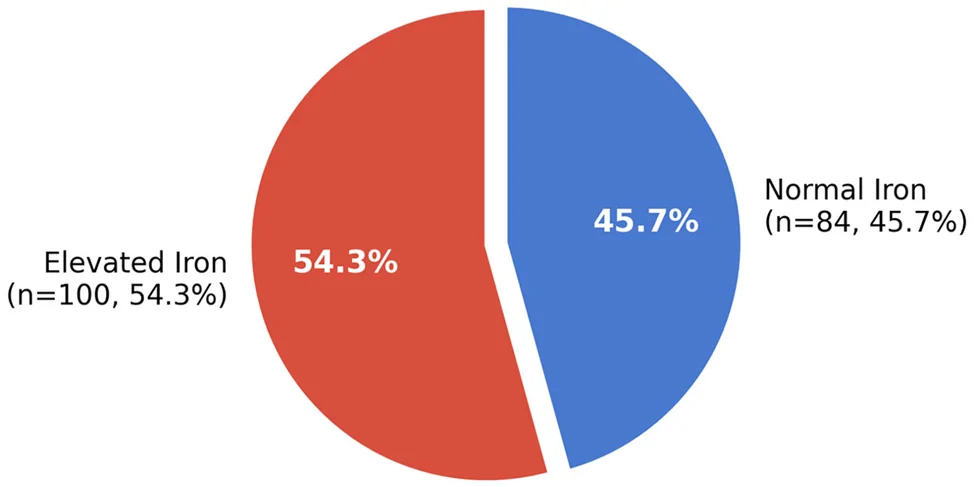
Distribution of serum iron status in the total study population (n=184).

**Figure 2:**
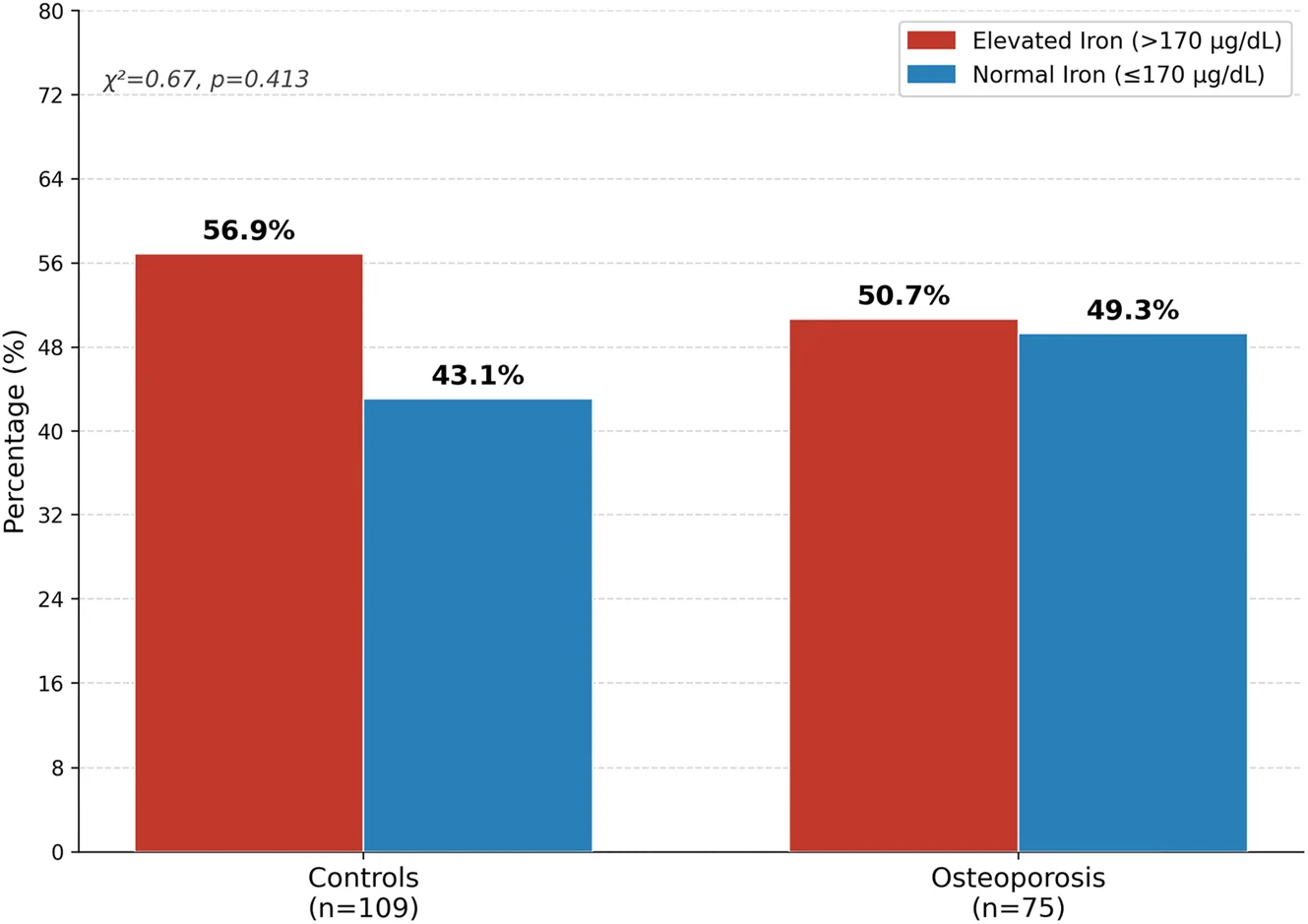
Proportion of participants with normal vs. elevated serum iron by osteoporosis status.

Table 1 summarises the demographic, clinical, and laboratory characteristics of both groups. Osteoporotic women were significantly older (mean age 58.5 ± 8.2 years) than controls (53.5 ± 5.1 years; p<0.001) and had a significantly lower mean BMI (30.4 ± 5.7 kg/m^2^ vs. 34.0 ± 6.1 kg/m^2^; p<0.001). Years since menopause were also significantly greater in the osteoporosis group (p<0.001). As expected, BMD was significantly lower in the osteoporosis group at both the lumbar spine and left femur (p<0.001). Fasting glucose and insulin were significantly higher in controls (p=0.03 and p=0.04, respectively), while HDL-cholesterol was significantly elevated in the osteoporosis group (p=0.01) and triglycerides were higher in controls (p=0.04). Notably, serum 25-hydroxyvitamin D levels were unexpectedly higher in osteoporotic women (p=0.03); this finding may reflect unmeasured vitamin D supplementation and should be interpreted cautiously. Bone turnover markers (BsALP, osteocalcin, NTx, β-CrossLaps, P1NP) and iron indices (serum iron, TIBC, TSAT%, transferrin) were not significantly different between groups, consistent with the primary adjusted analysis showing no significant association between serum iron status and osteoporosis.

**Table 1:** Demographic, clinical, and laboratory characteristics of the study population.

Parameters	Control (n=109)	Osteoporosis (n=75)	p-Value
Age, years	53.5 ± 5.1	58.5 ± 8.2	<0.001
Body mass index, kg/m^2^	34.0 ± 6.1	30.4 ± 5.7	<0.001
Waist-to-hip ratio	0.9 ± 0.1	0.9 ± 0.1	0.08
Systolic BP, mmHg	124.6 ± 19.6	123.1 ± 16.7	0.62
Diastolic BP, mmHg	74.8 ± 11.0	73.5 ± 9.7	0.45
Age of menarche, years	13.3 ± 1.3	13.3 ± 1.6	0.86
Years of menopause	5.0 (3.0–8.0)	10.0 (4.0–19.0)	<0.001
BMD spine (T-score)	−0.2 (−0.6–0.2)	−3.0 (−3.3 to −2.7)	<0.001
BMD dual femur left (T-score)	0.7 (−0.3–1.1)	−1.3 (−2.0 to −0.6)	<0.001
Glucose, mmol/L	7.7 ± 3.4	6.6 ± 3.0	0.03
Insulin, pg/mL #	226.3 (149.4–501.0)	174.3 (35.1–492.6)	0.04
Triglycerides, mmol/L	1.9 ± 0.9	1.6 ± 0.7	0.04
Total cholesterol, mmol/L	5.0 ± 1.2	5.1 ± 1.0	0.61
HDL-cholesterol, mmol/L	1.2 ± 0.3	1.3 ± 0.4	0.01
LDL-cholesterol, mmol/L	3.0 ± 1.0	3.0 ± 0.8	0.44
Vitamin D, nmol/L	70.4 ± 33.8	82.3 ± 37.5	0.03
Calcium, mmol/L	2.3 ± 0.3	2.3 ± 0.2	0.54
Albumin, g/L	40.6 ± 5.3	39.5 ± 4.5	0.37
Corrected Ca, mmol/L	2.3 ± 0.2	2.3 ± 0.2	0.56
NTx, nmol/L	56.5 ± 16.4	59.6 ± 29.0	0.75
β-Crosslaps, ng/mL	0.01 (0.01–0.09)	0.07 (0.01–0.10)	0.36
P1NP, ng/mL	15.3 (7.0–33.6)	20.5 (6.6–43.6)	0.45
BsALP, µg/L	15.1 (11.2–18.6)	14.2 (11.3–20.9)	0.45
Osteoprotegerin, ng/mL	0.6 (0.3–0.8)	0.4 (0.3–0.8)	0.39
Osteocalcin, ng/mL	4.8 (1.9–9.8)	5.4 (1.9–14)	0.25
Osteopontin, ng/mL	0.8 (0.5–2.2)	0.6 (0.3–1.6)	0.06
Sclerostin, ng/mL	0.7 (0.4–1.9)	0.7 (0.4–1.7)	0.54
Serum iron, µg/dL	180 (120–280)	180 (130–250)	0.94
TIBC, µg/dL	390 (280–430)	390 (310–440)	0.81
TSAT, %	38.3 (27.3–66.0)	44.9 (30.6–65.5)	0.41
Transferrin, mg/L	657.2 ± 190.3	692.2 ± 243.3	0.563

Data are presented as mean ± SD, or median (IQR) for non-normally distributed variables (#). p-Values were calculated using the independent-samples t-test or Mann–Whitney U test, as appropriate. Bold p-values indicate statistical significance (p<0.05). BP, blood pressure; BMD, bone mineral density; NTx, N-terminal telopeptide; BsALP, bone-specific alkaline phosphatase; P1NP, procollagen type I N-terminal propeptide; TIBC, total iron-binding capacity; TSAT, transferrin saturation.

Table 2 presents Spearman correlation coefficients between serum iron and all biochemical and demographic parameters, reported for all participants combined and for each group separately. Across all participants, serum iron was significantly and positively correlated with TSAT% (r=0.97; p<0.001), reflecting the expected physiological relationship between serum iron and transferrin saturation. Additional significant positive correlations were observed with LDL-cholesterol (r=0.17; p<0.05), osteocalcin (r=0.17; p=0.02), and osteopontin (r=0.17; p=0.049). However, all observed correlation coefficients were weak (r<0.20) with the exception of corrected calcium (r=−0.31; p<0.05). These correlations were generally of weak-to-moderate magnitude, and their clinical and biological significance remains uncertain. Figure 3 depicts the scatter plot of osteocalcin vs. serum iron across all participants, visually illustrating the weak positive relationship between these two variables (Spearman r=0.17, p=0.02).

**Table 2:** Spearman correlation coefficients between serum iron and demographic, clinical, biochemical, and bone turnover parameters in all participants, controls, and women with osteoporosis.

Parameters	All participants (n=184) R	Control (n=109) R	Osteoporosis (n=75) R
Age, years	−0.02	0.03	−0.12
Body mass index, kg/m^2^	−0.14	−0.15	−0.14
Waist-to-hip ratio	−0.03	−0.07	0.01
Systolic BP, mmHg	−0.03	−0.05	0.01
Diastolic BP, mmHg	−0.03	−0.06	0.04
Age of menarche, years	−0.07	−0.01	−0.12
Years of menopause	−0.08	−0.10	−0.11
BMD spine (T-score)	−0.03	−0.07	−0.09
BMD dual femur left (T-score)	0.07	0.11	0.06
Glucose, mmol/L	−0.07	−0.05	−0.10
Insulin, pg/mL	0.11	0.09	0.10
Triglycerides, mmol/L	−0.02	−0.10	0.11
Total cholesterol, mmol/L	0.11	0.06	0.20
HDL-cholesterol, mmol/L	−0.07	−0.15	0.06
LDL-cholesterol, mmol/L	0.17^a^	0.18	0.16
Vitamin D, nmol/L	−0.10	−0.01	−0.24
Calcium, mmol/L	−0.23	−0.21	−0.22
Albumin, g/L	−0.08	0.02	−0.17
Corrected Ca, mmol/L	−0.31^a^	−0.34^a^	−0.26
NTx, nmol/L	0.03	−0.02	0.20
β-Crosslaps, ng/mL	−0.06	−0.07	0.01
P1NP, ng/mL	−0.10	−0.08	−0.09
BsALP, µg/L	0.12	0.02	0.26
Osteoprotegerin, ng/mL	−0.11	−0.14	−0.07
Osteocalcin, ng/mL	0.17^a^	0.19	0.17
Osteopontin, ng/mL	0.17^a^	0.23^a^	0.07
Sclerostin, ng/mL	0.03	0.09	−0.05
TIBC, µg/dL	−0.17^a^	−0.21^a^	−0.10
TSAT, %	0.97^b^	0.97^b^	0.97^b^
Transferrin, mg/L	0.13	0.16	0.09

^a^Data are presented as Spearman correlation coefficients (r). ^a^p<0.05; ^b^p<0.01. r indicates the Spearman rank correlation coefficient.

**Figure 3:**
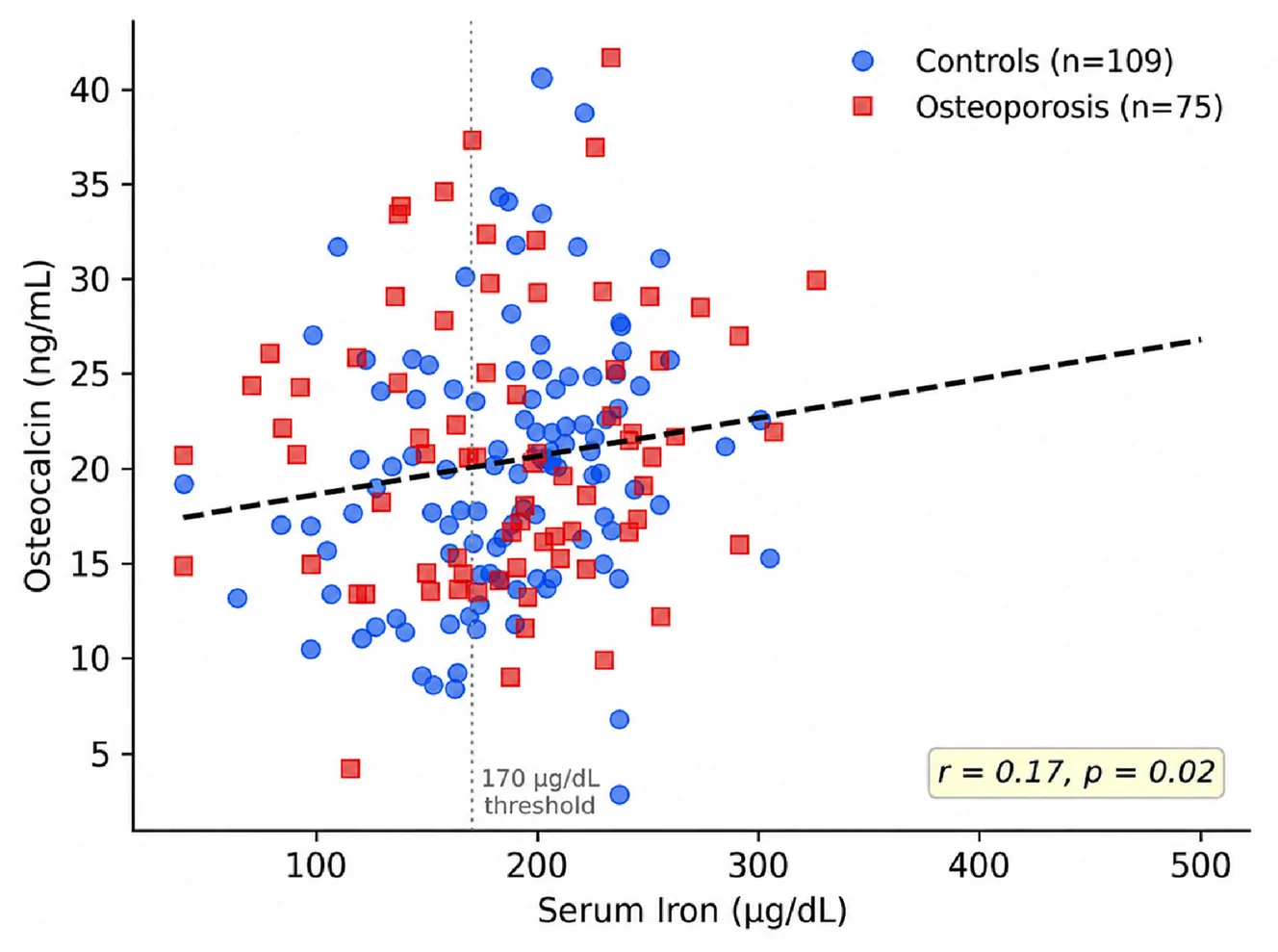
Scatter plot: association between osteocalcin and serum iron levels in control and osteoporosis groups.

Table 3 presents Spearman correlations of serum iron with all parameters stratified by iron status (normal vs. elevated). In the normal iron group, TSAT% was strongly and positively correlated with serum iron (r=0.74; p<0.01), as expected. In the elevated circulating iron group, the correlation between TSAT% and serum iron remained significant (r=0.75; p<0.01), and TIBC was significantly inversely correlated with serum iron (r=−0.47; p=0.01). No significant correlations with bone turnover markers were identified within either iron status stratum, suggesting that the weak correlations observed in Table 2 may reflect overall variation in serum iron levels across the cohort rather than iron status category alone.

**Table 3:** Spearman correlation coefficients between serum iron and demographic, clinical, biochemical, and bone turnover parameters according to serum iron status.

Parameters	Normal iron (50–170 μg/dL) R	Normal iron (50–170 μg/dL) p-Value	Elevated iron (>170 μg/dL) R	Elevated iron (>170 μg/dL) p-Value
Age, years	−0.06	0.64	−0.17	0.12
Body mass index, kg/m^2^	−0.05	0.71	0.00	0.98
Waist-to-hip ratio	−0.07	0.60	0.03	0.80
Systolic BP, mmHg	−0.02	0.85	−0.12	0.31
Diastolic BP, mmHg	−0.21	0.09	−0.01	0.92
Age of menarche	0.01	0.93	−0.02	0.88
Years of menopause	−0.06	0.65	−0.09	0.45
BMD spine (T-score)	−0.13	0.30	0.04	0.73
BMD dual femur left (T-score)	−0.13	0.30	0.15	0.19
Glucose, mmol/L	0.11	0.35	−0.04	0.70
Insulin, pg/mL	−0.11	0.37	0.10	0.39
Triglycerides, mmol/L	−0.02	0.87	0.02	0.84
Total cholesterol, mmol/L	0.15	0.22	0.03	0.76
HDL-cholesterol, mmol/L	−0.01	0.92	−0.03	0.80
LDL-cholesterol, mmol/L	0.22	0.07	0.04	0.75
Vitamin D, nmol/L	0.00	0.98	−0.15	0.19
Calcium, mmol/L	0.22	0.31	−0.09	0.60
Albumin, g/L	0.29	0.16	0.18	0.32
Corrected Ca, mmol/L	0.10	0.63	−0.27	0.11
NTx, nmol/L	0.10	0.87	−0.16	0.62
β-Crosslaps, ng/mL	0.14	0.64	−0.05	0.79
P1NP, ng/mL	0.19	0.53	−0.26	0.16
BsALP, µg/L	−0.01	0.93	−0.16	0.32
TIBC, µg/dL	−0.13	0.36	−0.47^a^	0.01
TSAT, %	0.74^b^	<0.01	0.75^b^	<0.01
Transferrin, mg/L	0.18	0.35	0.26	0.34

^b^Data are presented as Spearman correlation coefficients (r). p-Values correspond to Spearman rank correlation analysis. Normal iron: 50–170 μg/dL (n=84); elevated iron: >170 μg/dL (n=100). ^a^p<0.05; p<0.01. TIBC, total iron-binding capacity; TSAT, transferrin saturation.

Table 4 compares clinical and biochemical parameters between the control and osteoporosis groups, stratified by iron status. Osteoporotic women were significantly older than controls in both the normal iron (p<0.001) and elevated iron (p<0.001) subgroups. BMD at the lumbar spine and left femur was significantly lower in the osteoporosis group regardless of iron status (p<0.001 for all comparisons), confirming the primary diagnostic distinction between groups. Within the normal iron subgroup, serum iron was marginally higher in osteoporotic women compared to controls (p=0.051); this borderline result did not reach conventional statistical significance and should be interpreted with caution. Figure 4 illustrates BsALP levels by iron status in control and osteoporosis groups. A borderline non-significant trend toward higher BsALP was observed in osteoporotic women with elevated circulating iron compared to controls with elevated iron (p=0.056); given the absence of correction for multiple comparisons, this finding should be considered exploratory and hypothesis-generating only.

**Table 4:** Comparison of demographic, clinical, biochemical, and bone turnover parameters between control and osteoporosis groups stratified by serum iron status.

Parameters	Normal iron (Fe 50–170 μg/dL) control (n=47)	Normal iron (Fe 50–170 μg/dL) osteoporosis (n=37)	p-Value	Elevated iron (Fe >170 μg/dL) control (n=62)	Elevated iron (Fe >170 μg/dL) osteoporosis (n=38)	p-Value
Age, years	53.1 ± 4.2	58.0 ± 9.3	0.012	53.1 ± 5.1	58.7 ± 7.6	0.001
BMI, kg/m^2^	35.0 ± 4.9	31.1 ± 5.6	0.015	34.0 ± 6.3	29.7 ± 5.1	0.288
Waist-to-hip ratio	0.9 ± 0.1	0.9 ± 0.1	0.156	0.9 ± 0.1	0.9 ± 0.1	0.783
Systolic BP, mmHg	126.1 ± 17.7	123.5 ± 19.3	0.608	122.7 ± 20.6	125.6 ± 14.0	0.483
Diastolic BP, mmHg	75.5 ± 11.0	73.2 ± 10.1	0.243	74.6 ± 11.1	73.8 ± 9.8	0.713
Age of menarche, years	13.4 ± 1.5	13.5 ± 1.4	0.697	13.3 ± 1.3	13.3 ± 1.5	0.717
Years of menopause #	4.0 (4.0–7.0)	9.0 (4.0–19.5)	0.025	4.5 (2.0–7.0)	8.0 (4.0–12.0)	0.005
BMD spine (T-score)	−0.1 (−0.6–0.4)	−2.9 (−3.2 to −2.7)	<0.001	−0.3 (−0.7–0.2)	−3.1 (−3.5 to −2.6)	<0.001
BMD dual femur (T-score)	0.6 (−0.6–1.3)	−1.4 (−2.0 to −0.6)	<0.001	0.7 (0.1–1.1)	−1.1 (−1.9 to −0.6)	<0.001
Glucose, mmol/L	8.0 ± 3.2	6.4 ± 2.3	0.027	7.3 ± 2.8	6.7 ± 3.2	0.317
Insulin, pg/mL	265.3 (150.4–563.2)	151.4 (17.9–232.0)	0.028	346.8 (171.2–506.2)	180.2 (34.9–697.9)	0.171
Triglycerides, mmol/L	2.0 ± 0.9	1.5 ± 0.6	0.032	1.8 ± 1.0	1.7 ± 0.9	0.939
Total CHL, mmol/L	5.0 ± 1.1	5.0 ± 1.0	0.847	5.0 ± 1.1	5.3 ± 1.0	0.119
HDL-CHL, mmol/L	1.2 ± 0.3	1.3 ± 0.4	0.105	1.1 ± 0.3	1.3 ± 0.3	0.061
LDL-CHL, mmol/L	2.8 ± 0.8	3.0 ± 0.9	0.140	3.0 ± 0.9	3.2 ± 0.8	0.631
Vitamin D, nmol/L	67.2 ± 35.4	90.4 ± 41.7	0.021	66.8 ± 31.9	73.8 ± 32.2	0.976
Calcium, mmol/L	2.4 ± 0.3	2.4 ± 0.3	0.997	2.2 ± 0.2	2.3 ± 0.1	0.121
Albumin, g/L	42.2 ± 6.3	40.3 ± 3.5	0.473	38.7 ± 3.9	38.8 ± 5.1	0.700
Corrected Ca, mmol/L	2.4 ± 0.2	2.4 ± 0.3	0.724	2.2 ± 0.1	2.3 ± 0.1	0.286
NTx, nmol/L	53.2 ± 20.2	57.1 ± 50.5	0.931	59.4 ± 17.1	56.2 ± 39.3	0.940
β-Crosslaps, ng/mL	0.0 (0.0–0.1)	0.1 (0.0–0.1)	0.758	0.0 (0.0–0.1)	0.1 (0.0–0.1)	0.522
P1NP, ng/mL	14.1 (8.0–33.6)	28.8 (6.6–51.1)	1.000	16.4 (5.0–32.3)	23.7 (16.0–43.6)	0.291
BsALP, µg/L	16.1 (11.2–18.9)	13.1 (10.7–18.3)	0.638	13.8 (10.5–16.3)	18.1 (13.3–23.6)	0.056
Osteoprotegerin, ng/mL	0.5 (0.2–0.7)	0.3 (0.3–0.7)	0.729	0.6 (0.4–0.8)	0.5 (0.3–0.8)	0.739
Osteocalcin, ng/mL	2.7 (1.7–6.5)	4.9 (1.7–14.0)	0.249	5.9 (2.6–10.4)	7.4 (2.7–14.8)	0.716
Osteopontin, ng/mL	0.7 (0.4–2.3)	0.6 (0.3–1.6)	0.272	1.6 (0.6–2.7)	0.6 (0.2–2.5)	0.092
Sclerostin, ng/mL	0.8 (0.4–2.3)	0.7 (0.3–0.9)	0.224	1.0 (0.5–1.8)	1.3 (0.3–2.3)	0.489
Serum iron, µg/dL	112.5 (82.1–139.5)	129.2 (95.1–153.6)	0.051^a^	264.9 (199.0–361.9)	237.3 (204.1–327.4)	0.179
TIBC, µg/dL	410 (270–430)	390 (300–450)	0.858	380 (300–420)	390 (310–450)	0.715
TSAT, %	31.5 (19.5–37.8)	32.8 (25.7–46.8)	0.319	67.7 (47.0–96.0)	57.4 (45.6–90.4)	0.683
Transferrin, mg/L	653.0 ± 254.4	639.7 ± 213.1	0.88	679.1 ± 151.8	639.1 ± 192.8	0.67

Data are presented as mean ± SD, or median (IQR). p-Values were calculated using the independent samples t-test or Mann–Whitney U test, as appropriate. Normal iron: 50–170 μg/dL; elevated iron: >170 μg/dL. BMI, body mass index; BMD, bone mineral density; NTx, N-terminal telopeptide; BsALP, bone-specific alkaline phosphatase; TIBC, total iron-binding capacity; TSAT, transferrin saturation.

**Figure 4:**
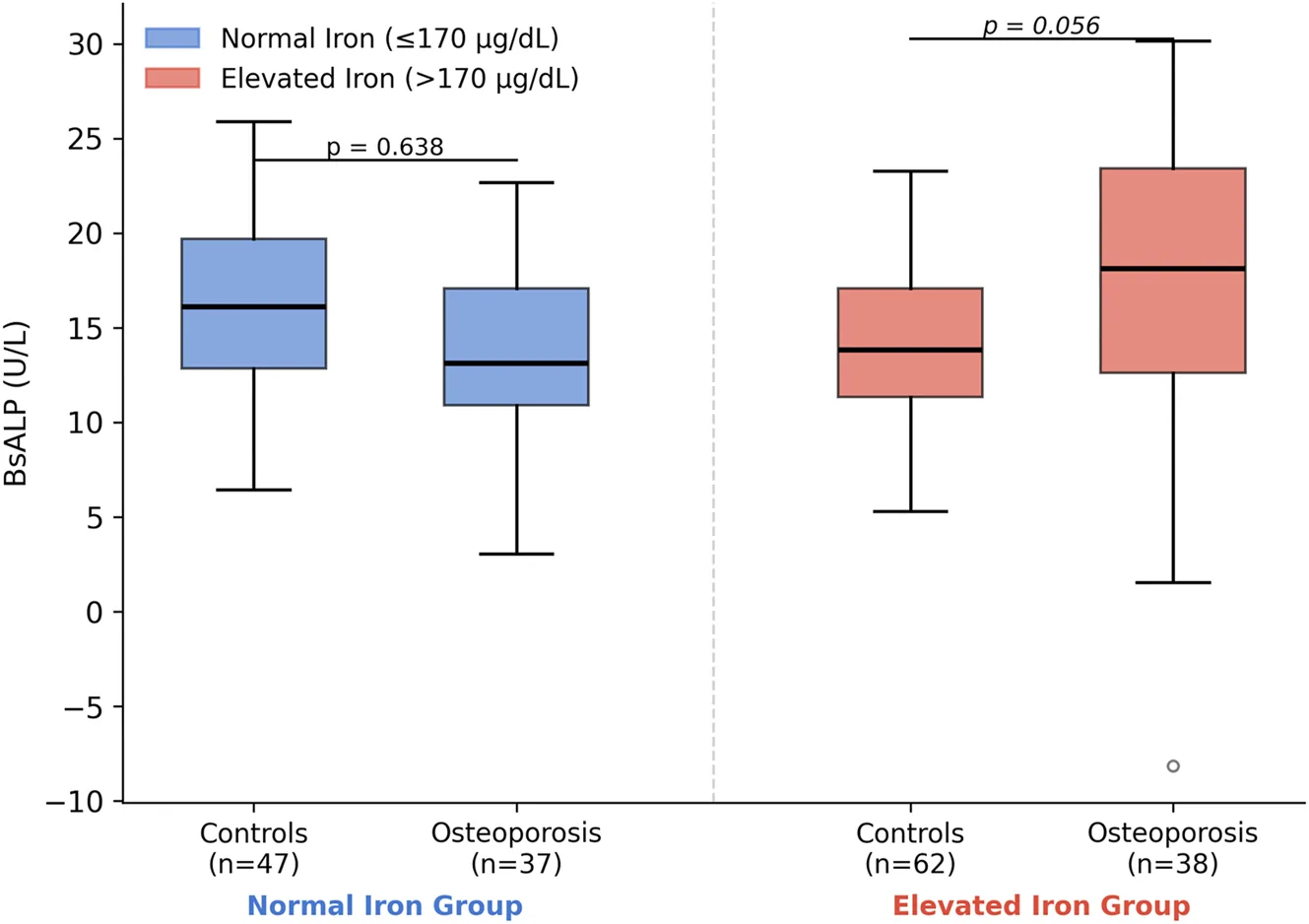
BsALP levels by serum iron status in control and osteoporosis groups.

Table 5 compares parameters according to iron status (normal vs. elevated) across all participants, controls, and osteoporotic women separately. Overall, participants with elevated circulating iron had significantly lower serum calcium (p=0.03) and albumin (p=0.04) compared with those with normal iron. Conversely, participants with elevated circulating iron had significantly higher osteocalcin (p=0.04), serum iron (p<0.01), and TSAT% (p<0.01). When examined within each group separately, no bone turnover marker reached statistical significance in either the control or osteoporosis subgroup after stratification by iron status, suggesting that the overall significant differences were driven by the combined sample rather than by group-specific effects. Figure 5 displays serum iron concentrations by iron status group across controls and osteoporotic women, confirming the expected and significant separation between normal and elevated iron subgroups (p<0.01) and illustrating the absence of a statistically significant difference in serum iron between the control and osteoporosis groups within each iron status stratum.

**Table 5:** Comparison of demographic, clinical, biochemical, and bone turnover parameters according to serum iron status in all participants and stratified by osteoporosis status.

Parameters	All participants Fe ≤170 (n=84)	All participants Fe >170 (n=100)	All p-Value	Controls Fe ≤170 (n=47)	Controls Fe >170 (n=62)	Controls p-Value
Age, years	55.8 ± 7.7	55.2 ± 6.5	0.55	53.1 ± 4.2	53.1 ± 5.1	0.98
BMI, kg/m^2^	33.1 ± 5.7	32.5 ± 6.3	0.52	35.0 ± 4.9	34.0 ± 6.3	0.34
Waist-to-hip ratio	0.9 ± 0.1	0.9 ± 0.1	0.88	0.9 ± 0.1	0.9 ± 0.1	0.82
Years of menopause	6.0 (3.0–14.0)	6.0 (3.0–11.0)	0.91	4.0 (4.0–7.0)	4.5 (2.0–7.0)	0.76
BMD spine (T-score)	−1.2 (−2.8–0.0)	−1.0 (−2.9–0.0)	0.79	−0.1 (−0.6–0.4)	−0.3 (−0.7–0.2)	0.53
BMD dual femur (T-score)	−0.1 (−1.0–0.9)	0.1 (−0.9–1.0)	0.50	0.6 (−0.6–1.3)	0.7 (0.1–1.1)	0.64
Vitamin D, nmol/L	77.3 ± 39.7	69.7 ± 32.0	0.21	67.2 ± 35.4	66.8 ± 31.9	0.95
Calcium, mmol/L	2.4 ± 0.3	2.2 ± 0.2	0.03	2.4 ± 0.3	2.2 ± 0.2	0.04
Albumin, g/L	41.5 ± 5.3	38.8 ± 4.4	0.04	42.2 ± 6.3	38.7 ± 3.9	0.07
NTx, nmol/L	54.8 ± 29.1	58.9 ± 19.5	0.73	53.2 ± 20.2	59.4 ± 17.1	0.60
BsALP, µg/L	13.4 (11–19)	15.5 (12–21)	0.12	16.1 (11–19)	13.8 (10–16)	0.62
Osteocalcin, ng/mL	3.1 (1.7–8.7)	5.9 (2.7–12.2)	0.04	2.7 (1.7–6.5)	5.9 (2.6–10.4)	0.03
Osteopontin, ng/mL	0.6 (0.3–1.6)	0.8 (0.5–2.6)	0.08	0.7 (0.4–2.3)	1.6 (0.6–2.7)	0.10
Sclerostin, ng/mL	0.7 (0.4–1.7)	1.0 (0.4–2.0)	0.20	0.8 (0.4–2.3)	1.0 (0.5–1.8)	0.56
Serum iron, µg/dL	121 (92–142)	250 (201–355)	<0.01	112 (82–140)	265 (199–362)	<0.01
TIBC, µg/dL	400 (280–440)	380 (300–430)	0.93	410 (270–430)	380 (300–420)	0.89
TSAT, %	31.6 (24–45)	61.7 (46–96)	<0.01	31.5 (19.5–38)	67.7 (47–96)	<0.01
Transferrin, mg/L	648.1 ± 233.1	654.1 ± 654.1	0.93	653.0 ± 254.4	679.1 ± 151.8	0.82

Data are presented as mean ± SD, or median (IQR). p-Values were calculated using the independent samples t-test or Mann–Whitney U test, as appropriate. Normal iron: 50–170 μg/dL; elevated iron: >170 μg/dL. BMI, body mass index; BMD, bone mineral density; NTx, N-terminal telopeptide; BsALP, bone-specific alkaline phosphatase; TIBC, total iron-binding capacity; TSAT, transferrin saturation.

**Figure 5:**
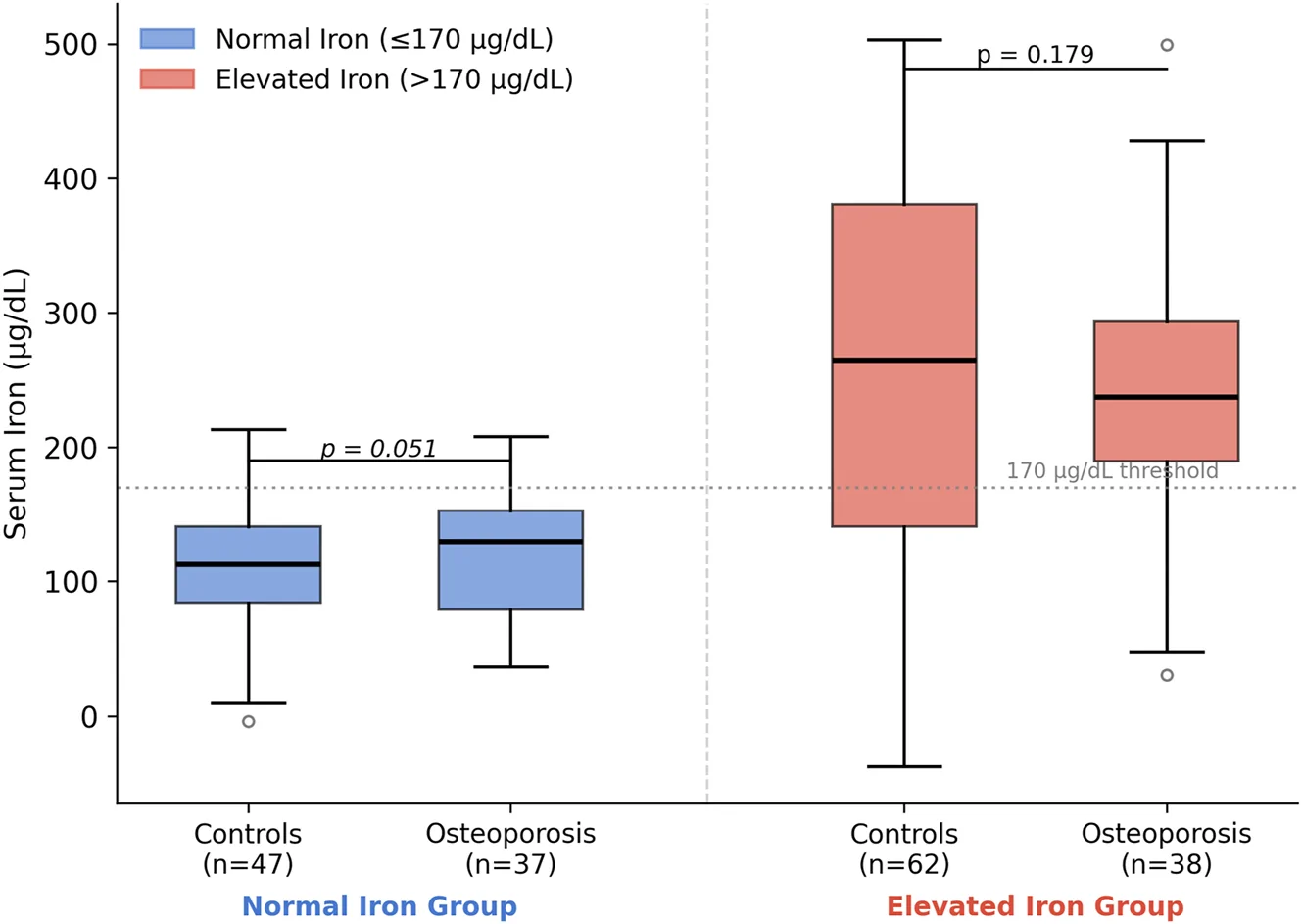
Serum iron levels by iron status group in control and osteoporosis subgroups.

Table 6 presents the primary pre-specified adjusted analysis: binary logistic regression of osteoporosis risk on serum iron exposure, modelled as both quartiles and tertiles and adjusted for age and BMI. This analysis constitutes the principal finding of the study. No statistically significant association was observed between serum iron and osteoporosis risk in any quartile or tertile (all adjusted p>0.18; all 95 % confidence intervals crossing 1.0). These results suggest that elevated serum iron, as defined by the local laboratory reference range (>170 μg/dL), was not independently associated with osteoporosis after adjustment for age and BMI in this cohort of postmenopausal Saudi women. The unadjusted and exploratory associations reported in Tables 2–5 and Figures 3–5 must therefore be interpreted in the context of this primary null adjusted finding and should not be used to infer causality or clinical significance.

**Table 6:** Binary logistic regression analysis of osteoporosis risk according to serum iron quartiles and tertiles.

Fe quartile/tertile	Unadjusted OR (95 % CI)	p-Value	Adjusted OR^a^ (95 % CI)	Adjusted p-Value
**Fe quartiles**				
**Fe tertiles**				
Quartile 1 (≤125.2 μg/dL)	Reference	–	Reference	–
Quartile 2 (125.3–180.1 μg/dL)	2.37 (0.94–6.00)	0.067	2.07 (0.72–5.94)	0.18
Quartile 3 (180.2–252.9 μg/dL)	1.92 (0.76–4.84)	0.17	1.37 (0.46–4.10)	0.58
Quartile 4 (≥253.0 μg/dL)	1.00 (0.39–2.58)	1.00	1.00 (0.34–3.03)	0.99
Tertile 1 (≤140.9 μg/dL)	Reference	–	Reference	–
Tertile 2 (141.0–217.2 μg/dL)	2.05 (0.93–4.52)	0.075	1.81 (0.71–4.59)	0.211
Tertile 3 (≥217.3 μg/dL)	1.03 (0.46–2.31)	0.938	1.20 (0.47–3.08)	0.699

^a^Adjusted for age and body mass index (BMI). Quartile 1 and tertile 1 served as the reference categories. OR, odds ratio; CI, confidence interval.

## Discussion

Bone is one of the major tissues that may be affected by alterations in iron metabolism [12], 13], 37]. Although some studies have reported associations between serum iron indices and bone metabolism, the direction, magnitude, and independence of these associations remain uncertain [38], [39], [40]. The present study examined the cross-sectional relationship between elevated circulating iron, bone mineral density (BMD), and bone turnover markers (BTMs) in postmenopausal Saudi women.

The aim of the present study was to investigate the association between serum iron status and osteoporosis in postmenopausal Saudi women. Osteoporotic women with serum iron levels within the normal range (50–170 μg/dL) exhibited marginally higher serum iron concentrations than controls. However, after adjustment for age and BMI, no statistically significant association was observed between serum iron status and osteoporosis risk (Table 6). This primary adjusted analysis suggests that previously reported unadjusted associations may be influenced by differences in age, body composition, or other confounding factors and should therefore be interpreted cautiously.

Two findings from Table 1 warrant discussion. First, serum 25-hydroxyvitamin D levels were significantly higher in osteoporotic women. This finding may reflect vitamin D supplementation following osteoporosis diagnosis and should therefore be interpreted cautiously, as supplementation history was unavailable. Second, BMI was significantly lower in osteoporotic women, which is consistent with the well-established inverse association between BMI and osteoporosis risk and has been widely reported in previous studies.

Several previous studies have reported associations between iron accumulation and bone loss among postmenopausal women [18], 21], [23], [24], [25], [26], [27, 41] and in animal models [28], [42], [43], [44], [45]. These studies observed that excess iron was associated with altered bone remodelling markers and reduced bone mineral density. However, the cross-sectional and retrospective nature of most human studies [14], 21], including the present study, precludes causal inference. Reverse causation and residual confounding by unmeasured factors, including ferritin, estradiol, inflammatory markers, and medication use, cannot be excluded [46], [47], [48].

Regarding BTMs, BsALP showed a borderline non-significant trend toward higher values in osteoporotic women with elevated circulating iron compared with controls with elevated circulating iron (p=0.056). Given the absence of statistical significance and the lack of corresponding changes in other BTMs, including NTx, β-CTX, P1NP, osteoprotegerin, and sclerostin, this finding should be considered exploratory. Experimental studies have generally suggested that excess iron suppresses osteoblast differentiation and alkaline phosphatase activity [49], [50], [51]; therefore, the observed trend should be interpreted cautiously and requires confirmation in larger prospective studies.

Another observation was the significantly higher osteocalcin concentration among women with elevated circulating iron (>170 μg/dL). In addition, serum iron showed a weak positive correlation with osteocalcin (r=0.17, p=0.02). Osteocalcin is a major non-collagenous protein of bone matrix and is commonly used as a marker of bone formation [52], [53], [54]. Previous studies have reported higher osteocalcin levels in postmenopausal compared with premenopausal women [50], 54], 55]. However, the clinical significance of this weak association remains uncertain.

Serum iron was weakly correlated with osteopontin in all participants (r=0.17, p=0.049). Osteopontin is an extracellular matrix protein secreted by both osteoblasts and osteoclasts and has been implicated in the regulation of bone remodelling [56], [57], [58]. Increased osteopontin concentrations have been linked to enhanced osteoclast activity and bone resorption [51], 59]. Similar to osteocalcin, the observed correlation was weak and should be interpreted cautiously.

Several biological mechanisms have been proposed to explain the potential relationship between iron metabolism and bone remodelling [28], [60], [61], [62]. Iron may contribute to reactive oxygen species generation through the Fenton reaction, potentially inhibiting osteoblast differentiation [49], 50], 63] and promoting osteoclast activity [64], 65]. Elevated iron has also been associated with impaired Wnt signalling in osteoblasts [66], 67] and increased adipogenic differentiation of mesenchymal stem cells [68], 69]. However, these mechanisms have been demonstrated primarily in experimental models, and their relevance to the weak cross-sectional associations observed in the present study remains uncertain. Importantly, the primary adjusted analysis (Table 6) demonstrated no independent association between serum iron status and osteoporosis after adjustment for age and BMI, indicating that the observed unadjusted correlations should be interpreted as exploratory findings rather than evidence of a causal relationship.

### Strengths and limitations

This study has several strengths, including the use of DXA-based assessment of BMD at the lumbar spine and left femur, evaluation of a comprehensive panel of bone turnover markers, and investigation of a clinically relevant and under-studied population of Saudi postmenopausal women. Nevertheless, several limitations should be acknowledged. First, the cross-sectional design precludes causal inference. Second, the serum iron threshold of >170 μg/dL was based on the upper limit of the local laboratory reference range and does not represent a validated clinical definition of iron overload. Although a sensitivity analysis using TSAT ≥45 % yielded directionally similar findings, only 12 participants met this criterion, limiting the precision of subgroup analyses. Third, serum ferritin measurements were unavailable, limiting the assessment of total body iron stores. Fourth, estradiol and FSH measurements were unavailable, limiting adjustment for hormonal factors that may influence bone metabolism. Fifth, the sample size may have limited the ability to detect modest associations. Sixth, medication history, including bisphosphonates, hormone replacement therapy, calcium/vitamin D supplementation, and iron supplementation, was not systematically recorded and may have influenced the observed findings. Seventh, transferrin concentrations were measured using an ELISA-based assay and are reported according to the manufacturer’s calibration units; therefore, direct comparison with values obtained using routine clinical chemistry platforms should be undertaken cautiously. Finally, the single-center design and inclusion of Saudi women only may limit the generalizability of the findings. Despite these limitations, the present study provides exploratory data on the relationship between serum iron status, bone mineral density, and bone turnover markers in postmenopausal Saudi women and may inform future prospective studies incorporating comprehensive iron and hormonal profiling to further clarify the relationship between iron metabolism and bone health in postmenopausal women.

## Conclusions

In this cross-sectional study of postmenopausal Saudi women, elevated circulating iron was common among study participants exhibiting serum iron levels above the predefined threshold. However, the primary adjusted analysis showed no statistically significant independent association between elevated circulating iron and osteoporosis risk after adjustment for age and BMI. Weak positive correlations were observed between serum iron and osteocalcin and osteopontin, while BsALP showed a borderline non-significant trend toward higher values in osteoporotic women with elevated circulating iron. These findings should be considered exploratory and do not support an independent association between elevated circulating iron and postmenopausal osteoporosis in this cohort. Future prospective studies incorporating comprehensive iron status assessment, hormonal profiling, and medication history are needed to further clarify the relationship between iron metabolism and bone health in postmenopausal women.

## Supplementary Material

Supplementary Material
