# Supplementary material for: Association of elevated circulating iron with bone mineral density and bone turnover markers in postmenopausal Saudi women with and without osteoporosis
Source: Open Med (Wars). 2026 Aug 31;21(1):20261507. doi: 10.1515/med-2026-1507 (PMC13693953; doi:10.1515/med-2026-1507)
Supplement: Supplementary file 1 [file j_med-2026-1507_suppl_001.docx]

**Association of Elevated Circulating Iron with Bone Mineral Density and Bone Turnover Markers in Postmenopausal Saudi Women with and without Osteoporosis**

**SUPPLEMENTARY DATA**

**Supplementary Notes**

**Supplementary Note 1. Statement on unavailability of serum ferritin and estradiol**

Serum ferritin was not measured in the original study protocol. This represents a recognised limitation of the present study, as ferritin is the single most informative circulating marker of total body iron stores and is required for the clinical diagnosis of iron overload. Stored serum samples are no longer available for retrospective ferritin measurement. Future studies should include ferritin, TSAT, and hepcidin measurements to provide a more complete characterisation of iron status.

Similarly, estradiol and FSH were not measured, precluding adjustment for endogenous estrogen status. As estrogen is a major determinant of bone turnover in postmenopausal women, its absence as a covariate limits the interpretation of iron–bone marker associations. Future studies should include hormonal markers to disentangle the effects of iron from those of estrogen deficiency.

**Supplementary Note 2. Post-hoc power calculation**

A post-hoc power analysis was performed using G*Power (version 3.1). Based on the study sample size of 184 participants (75 osteoporotic women and 109 controls) and a two-sided α level of 0.05, the achieved statistical power was estimated to be approximately 62%. The study was not powered a priori, and the modest sample size may have limited the ability to detect small-to-moderate associations. Consequently, the absence of statistically significant findings in the adjusted analyses should be interpreted cautiously, as it may reflect a true lack of association, limited statistical power, or both. Future larger prospective studies are needed to confirm these findings.
